# Extending protein-film electrochemistry across enzymology and biological inorganic chemistry to investigate, track and control the reactions of non-redox enzymes and spectroscopically silent metals

**DOI:** 10.1007/s00775-025-02105-0

**Published:** 2025-03-01

**Authors:** Clare F. Megarity, Ryan A. Herold, Fraser A. Armstrong

**Affiliations:** 1 Department of Chemistry, Manchester Institute of Biotechnology, University of Manchester, Manchester, M1 7DN UK; 2 Department of Chemistry and Biochemistry, University of California, San Diego, La Jolla, CA 92093 USA; 3 Department of Chemistry, University of Oxford, Oxford, OX1 3QR UK

**Keywords:** Catalysis, Protein-film electrochemistry, Electrochemical leaf, Enzyme cascade, Dehydrogenases, Kinases

## Abstract

Protein film electrochemistry has helped to unravel many complex reactivities of electron-transferring proteins and enzymes. A versatile descendant, the ‘Electrochemical Leaf’, offers new opportunities to extend electrochemical control to myriad enzymes that neither transfer electrons nor catalyse any redox reaction, including those dependent on spectroscopically limited, labile or other challenging metal ions. By embedding a cascade comprised of several enzymes—one of which electrochemically recycles NAD(P)(H), a second being a dehydrogenase—within a porous electrode formed from fused nanoparticles, the interconnected reactions are tightly channeled to transmit energy and information, rapidly and interactively. Under nanoconfinement, nicotinamide cofactors and cascade intermediates serve as specific current carriers, far beyond the electron itself.

**Graphical abstract:**

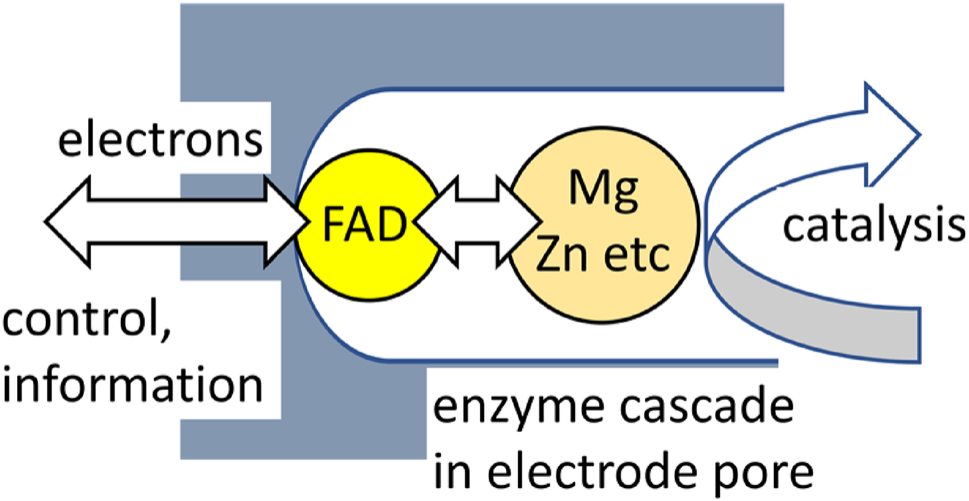

## Introduction

As anyone interested in biological inorganic chemistry is aware, a large proportion of enzymes require metal ions for their activity, directly as cofactors for catalysis, or indirectly as allosteric activators, or for structural integrity (and therefore activity) of the enzyme [1]. Redox-active metalloproteins have been very well studied—they usually involve non-labile metal ions, and at least one oxidation state is spectroscopically observable. In addition to structural and spectroscopic investigations, a suite of dynamic electrochemical methods termed protein-film electrochemistry (PFE), has provided unique complementary insight into mechanisms and functions of these proteins [2–5]. An emerging concept and platform known as the ‘Electrochemical Leaf’ (e-Leaf) affords the ability to extend PFE to study a much wider range of enzymes from all major classes—an important application being the real-time tracking of non-redox or labile metal ions, ligands, activators, and inhibitors, which have always presented a greater challenge to researchers [3].

**Fig. 1:**
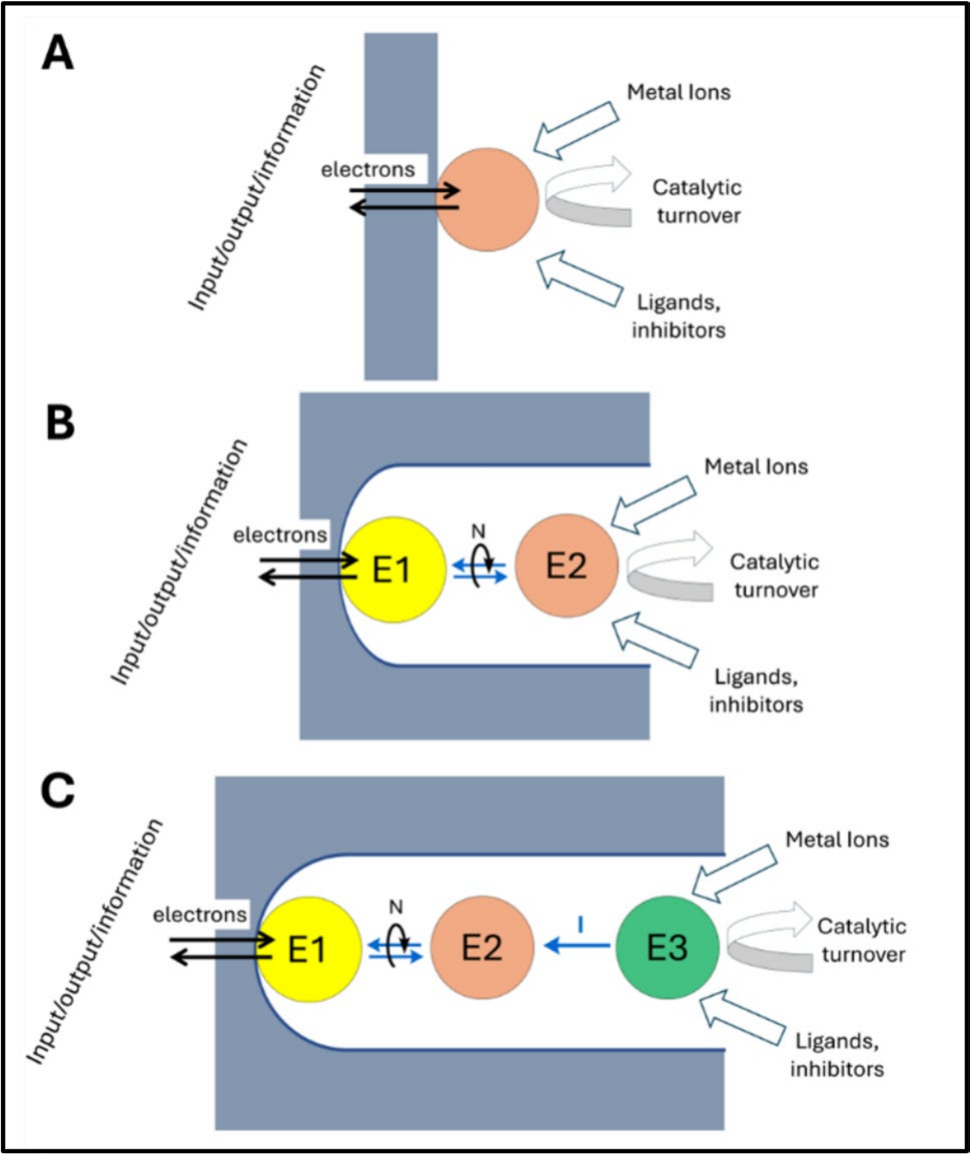
The evolution of protein-film electrochemistry. The electrode material is represented in gray. **A** Conventional model: one type of protein/enzyme adsorbed on a quasi-planar electrode. Information is obtained for those molecules oriented to allow efficient direct interfacial electron exchange. **B** Minimal e-Leaf: Electrocatalytic NAD(P)(H) transducing enzyme (E1) and dehydrogenase (E2) are loaded into the pores of an electrode. Under potential control, E1 undergoes direct electron exchange with the electrode and catalyzes the hydride-transfer interconversion between NADP^+^ and NADPH. E2 communicates with E1 via rapid nanoconfined recycling of the NAD(P)(H) which is represented as N. **C**. An extended e-Leaf: Further types of enzymes are included to build extended nanoconfined cascades. Current/information is transmitted as intermediate (I) passes from E3 to E2 (loss of intermediates to the bulk solution is suppressed due to the nanoconfined and crowded pore environment)

PFE was developed many years after it was discovered, in the late 1970s, that cytochrome *c* in solution exhibited direct (unmediated) quasi-reversible diffusion-controlled cyclic voltammetry at a metal oxide or modified gold electrode [6, 7]. Two independent pioneering papers challenged the popular dogma at the time, (a) that redox proteins are not active at electrodes, and (b) proteins denature at surfaces. It was important to note that most enzymes are far too large to diffuse rapidly and interact both transiently and productively with an electrode surface: thus, a solution-based enzyme should be effectively inactive as an electrocatalyst. The discoveries of the direct, unmediated electrocatalytic activities of cytochrome c peroxidase, succinate dehydrogenase and a hydrogenase, each tightly adsorbed on a carbon electrode, were important motivators—dramatically expanding the horizons [8–10]. Once it was accepted that redox-active protein molecules tightly adsorbed at a suitable electrode could display full retention of activity coupled to reversible, direct interfacial electron exchange, the way was open to develop PFE for investigating a wide range of electron-transferring metalloproteins. Requiring only minuscule quantities of material, PFE allowed exquisite control of a protein’s redox and catalytic properties, yielding insight—both thermodynamic and kinetic—from trailblazing investigations to detailed examination [2]. Throughout this paper we will refer to Fig. 1, where panel A represents the generic concept of conventional PFE.

Two advantages were immediately recognized, the first being that direct electron exchange with an *immobilized* redox-active center produces a well-defined and finite peak-type signal, unlike the broad waves and diffusive tails characteristic of solution voltammetry. The second aspect, particularly important for large enzymes, is that even if electron-exchange (‘non-turnover’) signals from internal redox sites are too faint to observe (due to coverage being very low), the enzyme turnover rate amplifies the response so much that the enzyme’s characteristics are still clearly revealed through the electrocatalytic current [10–12].

It is relevant to summarize the numerous examples of information that arose from early PFE investigations. It became possible to study unstable, vascillatory FeS centers or sites having extremely negative reduction potentials [13, 14]; cooperative two-electron transformations could be easily recognized and diagnosed, examples being cytochrome c peroxidase or arsenite oxidase [15, 16]; proton-gated electron transfer kinetics could be resolved using cyclic voltammetry at high scan rates (employing so-called ‘trumpet plots’) adapting theory introduced by Laviron [17–19]; unusual free-energy dependencies of the catalytic rate (*decreasing* with increasing driving force) became clearly visible (succinate dehydrogenase was the first example of an enzyme acting like a tunnel diode, displaying negative resistance) [10]; the reactions of hydrogenases could be analyzed in detail, establishing benchmarks for electrocatalysis [20]. In all of these cases, it was necessary for the redox-active site to engage in long-range electron tunnneling, as must occur in vivo, but now with the electrode surface as immediate donor/acceptor. Many enzymes were identified to be *reversible* electrocatalysts, requiring only a minimal overpotential to drive a reaction in either direction [21]. This measure of efficiency, previously observed only with Pt metals for H_2_ activation, suggested that electrochemical overpotential was among the drivers of early biomolecular evolution – oxidation and reduction being so essential for life that energy wastage needed to be minimized by numerous iterations of outer-shell and wider protein structure [22].

With a minuscule quantity of sample immobilized, the surrounding solution environment could be exchanged rapidly. Thus, for example, an exchangeable metal ion could be replaced by a different metal ion, exogenous ligands could be exchanged, the pH adjusted up and down and substrates could be removed as well as added. The outcomes of these interventions were immediately detectable. An electron-transfer protein or enzyme could be interrogated in all its redox states, with questions posed including the following: does a certain [3Fe-4S] cluster incorporate Fe, Zn, even Cu, Tl, or Pb?; does a redox-active metal coordinate a certain ligand?; and which oxidation states are involved in each case? [23]. Unlike conventional enzyme kinetics, the direction of catalytic turnover (rate being measured directly as current) could be switched immediately via the applied electrode potential, and the tendency of an enzyme containing a redox-active cofactor to operate more effectively in a particular direction (the inherent catalytic bias) could be quantified [24]. Others were taking enzyme electrochemistry in different directions, adapting for analysis/biosensors or scaling up for synthesis: for those applications it was less necessary for interfacial electron transfer to be direct, and small molecule mediators offered greater scope.

The e-Leaf, represented by the cascade maps shown in panels B and C of Fig. 1, extends PFE to enzymes that neither use long-range electron transfer nor even catalyze a redox reaction. Among the large fraction of all enzymes that bind a metal ion as a cofactor or activator, there are some 600 Mg^2+−^dependent enzymes and over 300 Zn^2+^-dependent enzymes in humans alone [25–27]. Numerous enzymes depend upon Mn^2+^, Ca^2+^ and other labile metals, and these may now be amenable to electrochemical study.

The e-Leaf is an operating system for handling enzyme cascades embedded within a mesoporous material: it is very easy to set up and use. A typical working device is comprised of a layer (1–10 µm thick) of indium tin oxide nanoparticles deposited by electrophoresis on a suitable electrode support, such as indium tin oxide glass, pyrolytic graphite edge or titanium foil. Important considerations are that the material is hydrophilic, electronically conducting, mechanically stable, and has pore dimensions (on the scale of 10–100 nm diameter) that favor inclusion of protein molecules varying widely in size and shape. The abilities to drive and monitor reaction rates in either direction, along with the fast response time arising from confinement and channeling, render the e-Leaf a powerful and interactive way to take a *systems* approach (observing how enzymes work in tandem and beyond) and address synthetic enzymatic reaction networks, an area of increasing interest across cell and synthetic biology [28, 29]. The essential points are: (a) the wide use by biology of nicotinamide cofactors for redox reactions; (b) fast, reversible electrocatalytic cycling of NAD(P)(H) by a flavoenzyme that is loaded into a porous electrode, transducing the current carrier from the electron (as in traditional PFE) to hydride, in the form of NAD(P)H; (c) the ability of the porous electrode material to host NAD(P)(H)-dependent dehydrogenases which recycle the NAD(P)(H) in a highly channeled manner in the nanoconfined and crowded pore environment; (d) the ability to accommodate further enzymes drawn from all major classes—dehydrogenases, hydrolases, transferases, ligases, isomerases (i.e., they need not be redox enzymes)—to create nanoconfined cascades [30–32].

The name ‘e-Leaf’ stems from the original discovery of the direct electrochemistry of ferredoxin NADP^+^ reductase (FNR), the small flavoenzyme found in green algae and chloroplasts which is responsible for recycling NADPH during photosynthesis, a key example being the Calvin cycle [33]. Now, instead of being driven (in one direction) by light, the reactions of FNR and other enzymes that connect to it in different ways in a leaf are driven (in both directions) by the electrode potential. Binding tightly within the hydrophilic pores of an indium tin oxide (ITO) electrode, FNR catalyses the reversible cycling of NADP(H), and serves as an excellent electron/hydride transducer. A C-terminal variant, Y354S, is selective for NAD^+^/NADH [34, 35].

A common feature of Fig. 1 is *confinement* – either on the surface of a planar electrode (A) or within a porous network (B and C). All cases involve a *target* enzyme—the subject of interest: for conventional PFE, this must be equipped for fast long-range electron transfer: but for the e-Leaf this restriction is unnecessary, as exchangeable cofactors and intermediates are now confined and channeled to function as specific current carriers within the electrode pores. The interactive analytical power of PFE (Fig. 1A) is extended to enzymes that use nicotinamide cofactors instead of electrons (Fig. 1B) and thence to all classes of enzymes that can be included in a cascade that has at least one NAD(P)(H) recycling step (Fig. 1C). Panels B and C in Fig. 1 are referred to as *cascade maps*: they depict enzymes that are ‘neighbors’, not in space but along a reaction sequence (close spatial proximity is probably not important) [36]. Through nanoconfinement (an appropriate term for the random tunnels and cavities generated by packed nanoparticles), and the natural high selectivities and activities of most enzymes, small-molecule intermediates have a higher probability of being captured and processed by the next enzyme than of escaping the porous layer [31]. An enzyme *upstream* of the dehydrogenase with regard to the sequence of reactions may determine the current, and by choice of conditions, this enzyme can be selected to be the *target*. Enzymes of all sizes enter the pores in what appears to be a spontaneous process [30]. While the protein molecules become physically trapped, NAD(P)(H) becomes partially trapped, and the probability of it encountering the active site of E2 upon its release from E1 (and vice versa), is massively increased in the nanoconfined and crowded environment where local enzyme concentrations can reach millimolar levels [37]. In contrast, small molecules and metal ions enter and leave relatively rapidly—thus, as with traditional PFE, oxidation and reduction directions can be switched virtually instantaneously, while species to be tested such as metal ions, sequestering chelators, ligands, inhibitors and potential catalytic substrates can easily be added and removed [38]. Although NAD(P)(H) is held quite tightly in the electrode pores (a high affinity for its concentrated enzymes being a likely contributing factor), a minimal concentration is maintained in solution to offset its limited escape [30, 37].

**Fig. 2:**
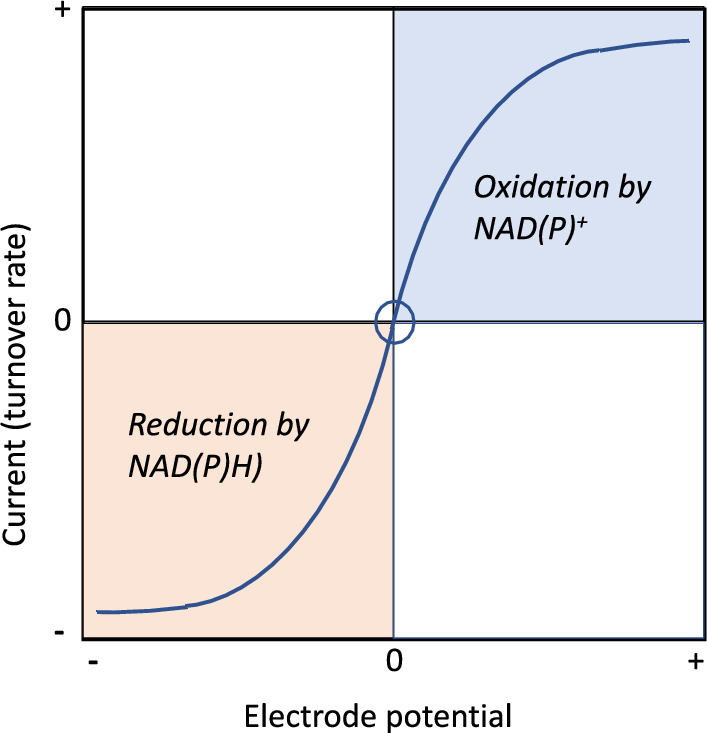
Generic representation of the voltammetry of an *O*/*R* redox system catalyzed by a non-electron-transferring enzyme that is nanoconfined and tightly coupled to rapid and reversible electrocatalytic NAD(P)(H) cycling in the same electrode pores. The origin (circled) is the potential at which no net current flows: it approximates to the formal potential of the *O*/*R* couple (referenced here as 0) when these are each present under a specific condition (usually one in which O and R are both present at equal concentrations). Operations such as addition or removal of metal ions, ligands, inhibitors, activators, may result in immediate responses to the rate when the current is monitored at a constant potential in either or both of the oxidation and reduction quadrants

It is helpful to refer to a generic voltammogram, appropriate to case B, in which a dehydrogenase catalyses the interconversion between oxidized and reduced forms (*O*/*R*) of its substrate. The voltammogram shown in Fig. 2 applies for reversible electrocatalysis (FNR electrochemistry is reversible in the Nernstian sense).

**Fig. 3:**
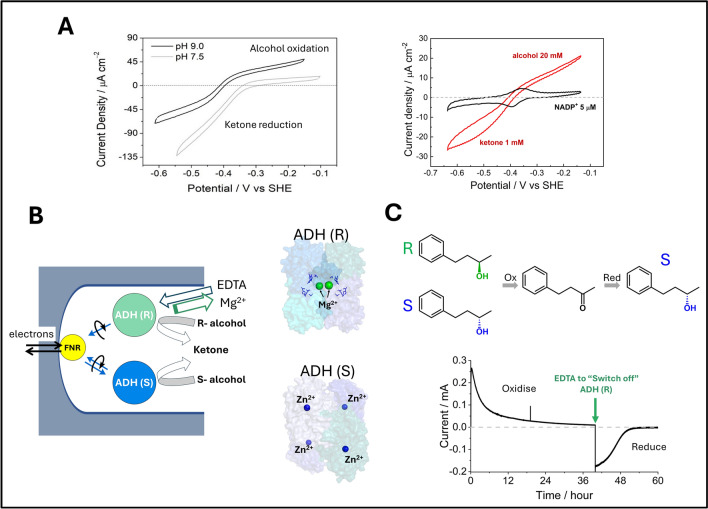
Electrochemical studies and exploitation of alcohol dehydrogenases (featuring Zn^2+^ or Mg^2+^). **A** *Left*: Cyclic voltammograms obtained for an ADH variant (W110A) from *Thermoanaerobacter ethanolicus* with equal concentrations of ketone and alcohol in solution. Electrocatalysis is bidirectional at pH 9 but strongly biased for reduction at pH 7.5. *Right*: Cyclic voltammograms for W110A recorded at pH 8 with a 20-fold excess of alcohol to ketone to compensate for the reductive bias. **B** *Left*: Scheme showing two ADH variants of opposite enantioselectivity loaded into the e-Leaf electrode; *Right*: corresponding structures (ADH (R) pdb code 6H07 and ADH (S) pdb code 7JNS). The ADH (R) from *Lactobacillus kefir* depends on two Mg^2+^ ions for structural integrity and activity; ADH (S) from *Thermoanaerobacter ethanolicus* has four tightly bound Zn^2+^ ions which are the catalytic centres. **C** Chronoamperometry experiment in which the electrode was used to deracemize a racemic mixture of secondary alcohols

Reversible NADP^+^/NADPH interconversion by the transducer FNR is slightly biased in the direction of NADP^+^ reduction (one reason being that the FNR-bound FAD cofactor has a slightly more negative reduction potential than NADP^+^) [33]. The exchangeable nicotinamide cofactor now behaves as a redox mediator, and we recall that potentiometric titrations of enzymes depend upon choosing mediators that have potentials close to the one being measured to facilitate equilibration through bidirectionality [39]. Accordingly, further along the cascade, the primary catalytic bias (the preference for oxidation vs reduction of reactants *O* and *R*) is determined by the difference in reduction potentials between the NAD(P)^+^/NAD(P)H and O/R redox couples, thus the bidirectionality is modulated by pH (− 30 vs − 60 mV per pH unit dependencies, respectively) and the *O*/*R* concentration ratio in solution, and is largely independent of the enzyme (see Fig. 3A for an experimental example). In contrast, the secondary bias reflects direction-dependent activation or inactivation, a simple example being product inhibition: this metric reports on the target enzyme.

The following examples are drawn from experiments that were not designed with the aim of explicitly studying the role and properties of metal ions interacting with proteins, but they indicate the scope for doing so. The case studies illustrate how information is obtained from each of the two main methods of applying protein-film electrochemistry: cyclic voltammetry, which displays how current (rate) varies with electrode potential (free energy); and chronoamperometry, which displays how current (rate) varies with time following a particular change in condition (injection of reactants, step change in potential).

### Examples of dehydrogenases (Zn^2+^ and Mg.^2+^-dependent)

Referring back to Fig. 1, the application of Case B is explained using the electrochemistry of NAD(P)-dependent alcohol dehydrogenases (ADH). Figure 3 shows an investigation in which two ADHs, differing in their structures and substrate selectivities (labelled as (ADH (R) and ADH(S) in Fig. 3B), were loaded into the electrode pores to study the stereoinversion or deracemization of a secondary alcohol by redox cycling (Fig. 3C).

Most alcohol dehydrogenases are Zn-enzymes [40]. Panel A shows cyclic voltammograms obtained for a Zn-ADH from *Thermoanaerobacter ethanolicus* catalyzing the interconversion between a secondary alcohol and a ketone. The voltammograms show how the thermodynamic catalytic bias—due to the difference in formal potentials for the NADP^+^/NADPH and alcohol/ketone couples—is manipulated by changing the pH (left) or ketone/alcohol ratio (right) [41]. This ADH is a tetramer, containing one tightly bound catalytic Zn^2+^ per monomer, and is selective for S-enantiomers. In contrast, an ADH (also a tetramer) from *Lactobacillus kefir* does not require Zn^2+^ as a catalytic cofactor but uses instead a Ser-Tyr-Lys catalytic triad: its activity, which is selective for R-enantiomers, depends on two labile Mg^2+^ that are responsible for preserving the active conformation [42]. The difference between the two enzymes was exploited to design an inverter/deracemizer for secondary alcohols, whereby the two ADH types (Zn^2+^  = S-selective; Mg^2+^  = R-selective) were loaded together in the electrode [43]. After exhaustively oxidizing the solution of alcohol to ketone by driving both enzymes with an oxidizing potential in the first half cycle, EDTA was added to sequester Mg^2+^ and inactivate the R-selective enzyme (the Zn^2+^ remained bound in the S-selective enzyme), so that when the potential was switched to a reducing value, the reduction half-cycle produced only the S-product (Fig. 3C).

Isocitrate dehydrogenases (IDH)—Mg^2+^-dependent enzymes catalysing the oxidative decarboxylation of D-isocitrate to 2-oxoglutarate (2OG) as shown below—are the focus of intense interest because 2OG is a central metabolite and frequent IDH mutations are associated with several cancers [44].
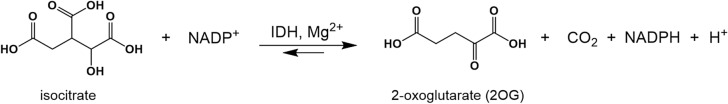


**Fig. 4:**
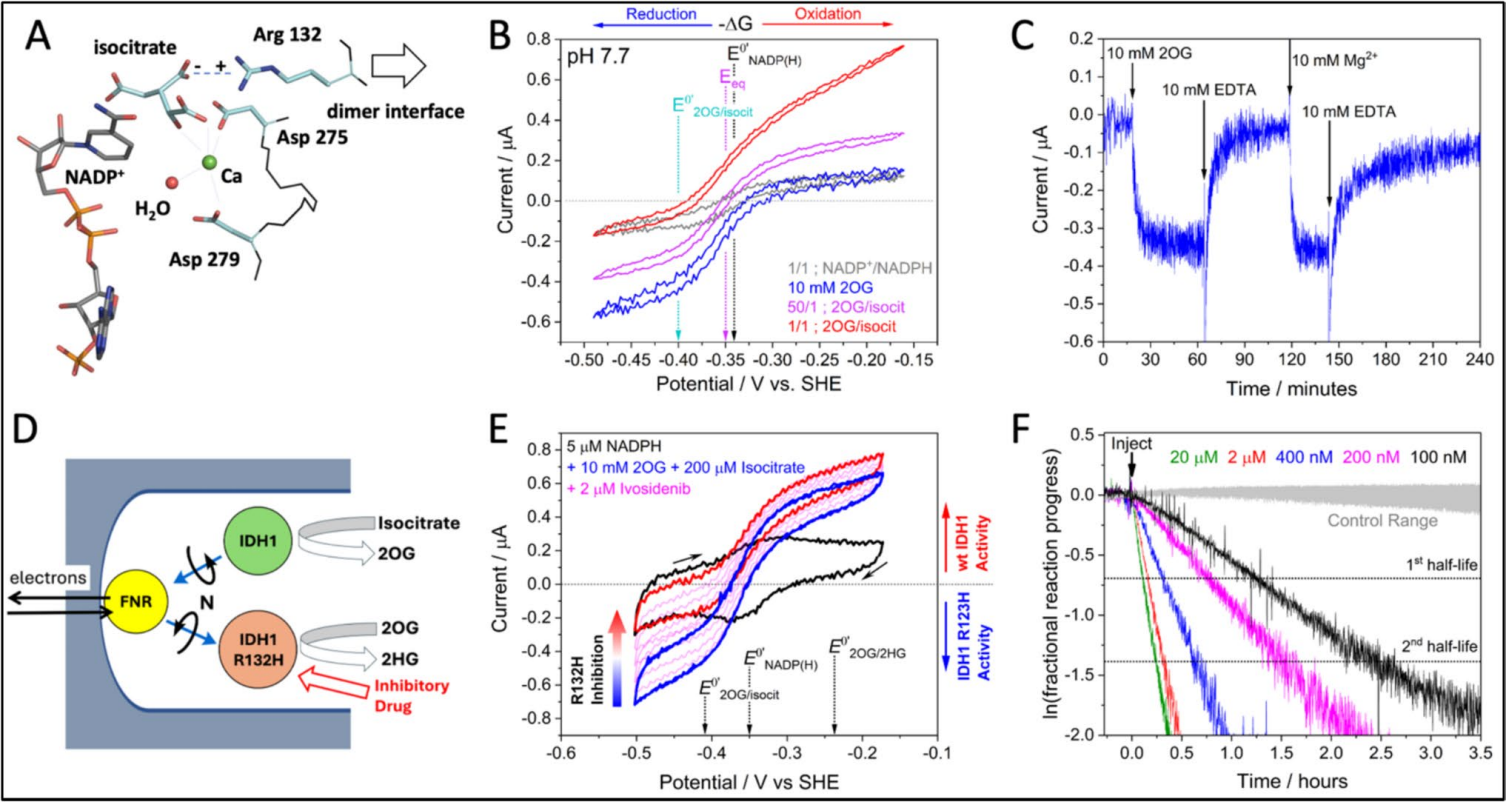
Electrochemical investigations of isocitrate dehydrogenases. **A** Active site of human wildtype IDH1 (1T0L) showing binding of isocitrate and NADP^+^ (Mg^2+^ is replaced by Ca^2+^ to give inactive enzyme). **B** Cyclic voltammograms showing catalysis by wildtype IDH1 under various conditions (solution contains 0.10 M NaHCO_3_). **C** Chronoamperometry experiment demonstrating the ease with which Mg^2+^ is removed from and replaced at the active site of the R132H variant. **D** Cascade map for the experiment in panel E showing wildtype IDH1 and IDH1 R132H loaded together alongside FNR, the action of which drives one IDH1 enzyme or the other depending on the applied potential. **E** An electrode co-loaded with wildtype IDH1 and IDH1 R132H under conditions in which both enzyme activities can be observed in the same experiment by cycling the electrode potential (wildtype IDH1 activity is observed as isocitrate oxidation while R132H activity is observed as 2OG reduction). Addition of ivosidenib to the reaction solution results in the slow, selective inhibition of IDH1 R132H while wildtype IDH1 activity is unaffected. **F** Linearized chronoamperometric traces showing pseudo first-order inhibition of IDH1 R132H at different concentrations of ivosidenib

Figure 4A shows the active site of human IDH1, which is localised in the cytoplasm and selective for NADP^+^ [45]. The panel indicates how molecules of NADP^+^ and isocitrate are arranged in the active site (one of two—the enzyme is a homodimer) but with the caveat that the Mg^2+^ has been replaced by Ca^2+^ to render the enzyme inactive. Panel B shows the cyclic voltammetry of IDH1 loaded in the e-Leaf and exposed to different concentrations of isocitrate and 2OG. At pH 7.7 the system is strongly biased in favor of 2OG formation (a 50:1 ratio is required to equalize the limiting currents in each direction), the bias following the rule mentioned above and applying for ADH [41].

The most important cancer-linked mutations of IDH1 occur at arginine-132 which interacts with one of the isocitrate carboxylates [44]. One notable example, R132H, has lost nearly all of its isocitrate oxidation activity, catalyzing instead the reduction (by NADPH) of 2OG to 2-hydroxyglutarate (2HG), a reaction displaying a > 2 orders of magnitude decrease in Mg^2+^ binding affinity [46].
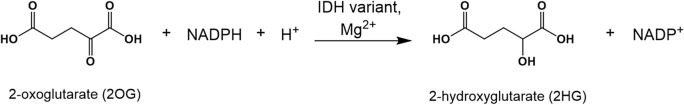


Among the drugs developed to inactivate these neomorphic (‘gain of function’) variants is ivosidenib, an allosteric inhibitor that binds slowly but tightly at the dimer interface, inhibiting the enzyme by locking it into an inactive conformation. The complex kinetics could be investigated in detail using the e-Leaf [38]. The process of inhibition is much slower than the binding and sequestration of Mg^2+^ by EDTA (Panel C) or its replacement by inhibitory Ca^2+^, each of which directly involve the active site [47]. Panel E shows a cyclic voltammetry experiment in which wildtype IDH1 (E2) and IDH1 R132H (E2’) were loaded into the same electrode (see Panel D). By exploiting the opposing dominant reactions catalyzed by each enzyme (oxidation of isocitrate for wildtype, vs reduction of 2OG for R132H), the activities of the two enzymes were monitored independently as the electrode potential was cycled. Introduction of ivosidenib inhibits the action of R132H while leaving wildtype IDH1 essentially unscathed. Such a ‘living CV’ provides an immediate glimpse of how a drug selects for its enzyme target, as it might occur in a cancer cell after being administered to a patient. In this case, the large decrease in the binding affinity of R132H for Mg^2+^ allows nanomolar concentrations of ivosidenib to *selectively* inhibit the cancer-associated variants—a conclusion that could only be *directly* confirmed by testing ivosidenib against a mixed population of active variant/wildtype IDH1 enzymes. Panel F shows linearized chronoamperometric (catalytic rate vs time) traces obtained with different concentrations of ivosidenib: the data were incorporated, along with those of many other similar measurements in which Mg^2+^ and 2OG were varied, to determine the kinetic mechanism of inhibition [38]. Points to note are: (i) traces are first order for two or more half-lives even at inhibitor concentrations commensurate with the lower practical limits of enzyme concentration in steady-state solution assays; (ii) the slow inhibition kinetics are revealed directly and efficiently, easily surpassing the capabilities of steady-state solution kinetics.

Analysis of the data, obtained over a wide range of 2OG and Mg^2+^ concentrations (and even very low levels of ivosidenib) revealed a wealth of information, including forward and reverse rate constants for inhibition [38]. It could be deduced that whereas the drug binds rapidly to the enzyme, its inhibitory action occurs in a slow second stage in which it locks into position at the dimer interface. The results highlight an important advantage over surface plasmon resonance, which would not discriminate between inhibitory and non-inhibitory binding.

### Examples of kinases: extending further out from electron transfer

Incorporating kinases into the e-Leaf opens up many new possibilities to link electrochemistry to processes that are even more remote from the FNR electron-transfer step (variations of Case C, Fig. 1). Carboxylic acid reductase (CAR) catalyses the reduction of a carboxylic acid to an aldehyde by NADPH and ATP, itself a complex of Mg^2+^ [48].
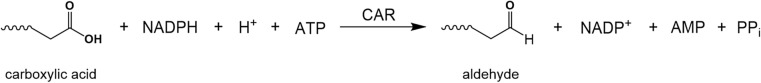


**Fig. 5:**
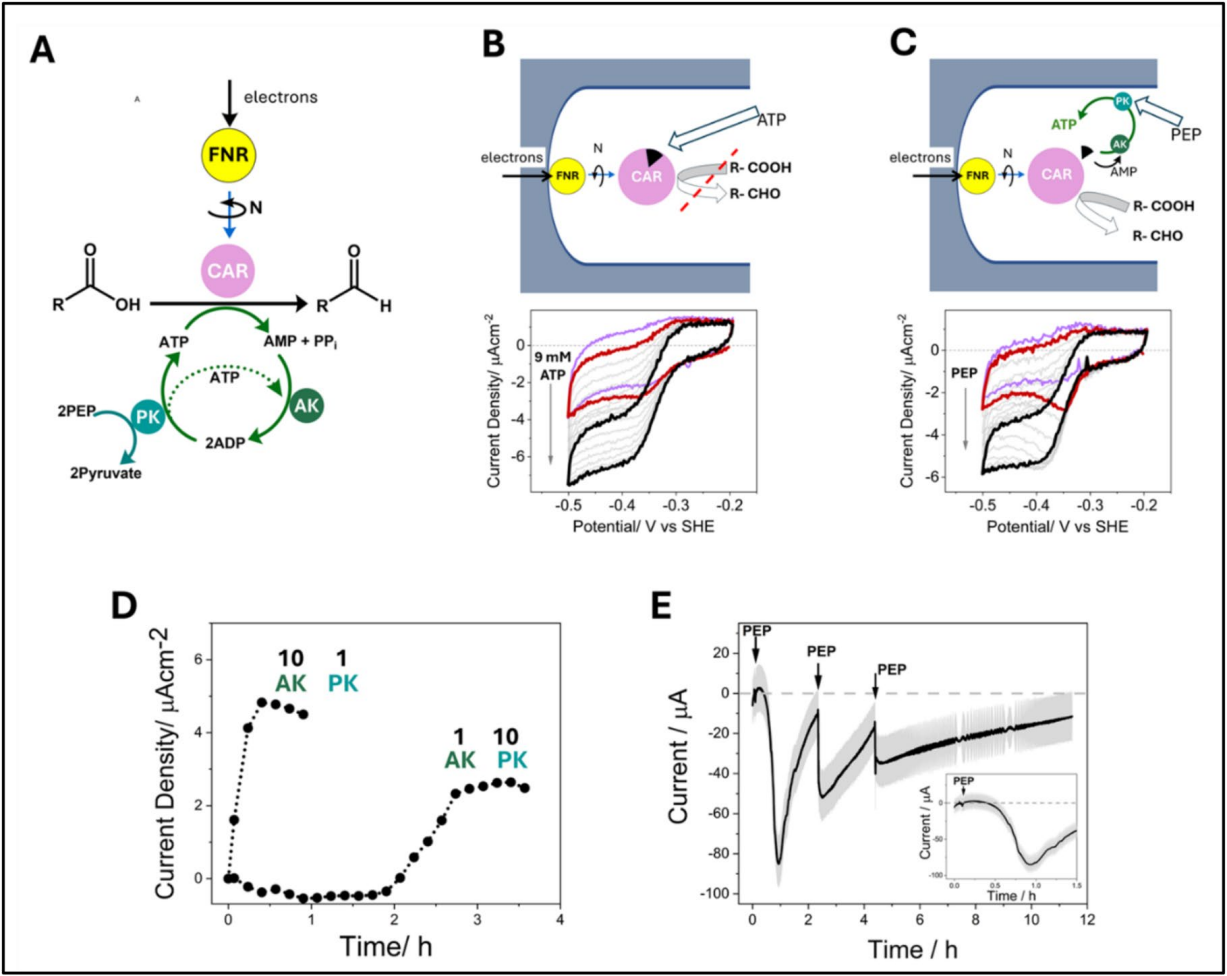
**A** Cascade design showing CAR driven electrochemically through connection to FNR and by chemical energy supplied as phosphoenolpyruvate (PEP) transduced by the kinase cascade (adenylate kinase, AK, and pyruvate kinase, PK). **B** Catalysis by CAR in the e-Leaf when supplied with ATP as a bulk reagent at high concentration. **C** Catalysis by CAR in the e-Leaf with a trace amount of ATP accumulated and recycled by the co-entrapped kinase pair; sequestration of the tightly bound AMP intermediate (black wedge) by AK is indicated. **D** The time dependency of the catalytic rate (current density) as ATP is accumulated by the kinase cascade at different ratios of each enzyme. The importance of AK in its role to sequester AMP is evidenced by the much shorter time taken to reach a maximum rate with the 10AK:1PK cascade ratio. **E** Fuel (PEP) depletion and replenishment during synthesis of cinnamaldehyde by CAR (driven by applying reducing potential) with accumulation and recycling of ATP from the trace amount contained in AMP, by the co-entrapped kinase cascade

Since use of the two cofactors is tightly coupled, the recycling of NADP^+^ by CAR effectively renders ATP ‘electroactive’, in that its involvement and the way it is recycled can be tracked rapidly. Through CAR, the electrocatalytic current reports on the actions of kinases that are co-loaded in the e-Leaf electrode, as represented in Fig. 5A [49]. The reaction is irreversible, and the catalytic reduction current commences at an electrode potential close to the NADP^+^/NADPH couple. The energy is provided electrochemically via FNR and chemically by direct addition of ATP (a high concentration is required) or, much more efficiently, by the phosphate donor phosphoenolpyruvate, PEP, in a sequence of reactions catalyzed by adenylate kinase (AK) and pyruvate kinase (PK) which together recycle ATP from AMP via ADP.

Comparing the respective voltammograms for time-dependent development of catalytic activity, it is clear that highly localized recycling (Fig. 5C) is far more effective than introducing ATP as a stoichiometric reactant (Fig. 5B). In fact, the kinase pair efficiently accumulates and recycles ATP from an exceptionally low trace level that is present in commercial preparations of AMP, the overall electrocatalytic rate being more sensitive to AK than PK (Fig. 5D). A plausible hypothesis for the special role of AK is based on the evidence that during its catalytic cycle CAR adopts a resting inactive state in which AMP is still bound: it is at this point that AK sequesters AMP directly upon its release from the enzyme, thus allowing the next cycle to commence (Fig. 5C). The rate is ultimately limited by kinase activity, as it is highly responsive to injections of the ‘fuel’ PEP (Fig. 5E). It is known that the activities of many kinases, including PK, depend on metal ions [50]. Thus, with suitable design of the cascade, the action of kinase activators and inhibitors would be immediately detected by chronoamperometry. The tight coupling of electron transfer to ATP hydrolysis is also reminiscent of nitrogenase, suggesting that the e-Leaf might offer another way to investigate this very complex enzyme system [51].

## Conclusions and outlook

The e-Leaf is easy to use once the kit is in place, and offers insight in two ways. The first advantage is for trailblazing, whereby the very interactive nature of the technique facilitates the screening of species likely to interact with a complex enzyme system. The second advantage is that the e-Leaf is able to generate quantitative data on affinities and kinetics with real-time decision-making and under conditions (maintenance of a steady-state under high enzyme concentration) that are not possible or practical with conventional enzyme studies. The power of electrochemical methods for controlling and measuring the action of enzymes through the current output is easily extended to non-redox enzymes that can be incorporated to become the targets. A further example, under review while this paper was being prepared, extends the concept of the target enzyme to urease, a Ni-containing hydrolase. In that work the catalytic current depended on the supply of NH_4_^+^, a substrate for glutamate dehydrogenase, generated in situ by the action of co-nanoconfined urease on urea [54]. A wider question is how well the synthetic environment resembles those which enzymes experience naturally; here we note that the protein molecules become very crowded (reaching millimolar concentration), unlike most conventional kinetic experiments but in more ways resembling conditions within living cells [37, 52, 53]. A potential practical advantage lies in the simple ability to corral different enzyme molecules of various sizes (a consequence of the random arrangement of pores produced by stacking nanoparticles) so that they attain high local concentrations yet sustain rapid steady-state turnover while substrate is supplied. It will be interesting to see if this feature opens up new opportunities for spectroscopy. Finally, PFE allows us to control and observe reactions and catalysis by electron-transport enzymes in a highly interactive way, and it is easy to *change direction, stop, resume, change condition, disperse product*: many of these features may now be extended to other enzymes and the cascades that they operate in.

## Data Availability

No datasets were generated or analysed during the current study.
